# A neuro decision-making approach for prioritizing circular economy criteria in sustainable smart cities

**DOI:** 10.1016/j.heliyon.2024.e40354

**Published:** 2024-11-13

**Authors:** Gang Kou, Hasan Dinçer, Serhat Yüksel, Fahd S. Alotaibi

**Affiliations:** a School of Business Administration, Faculty of Business Administration, Southwestern University of Finance and Economics, Chengdu, 611130, China; b The School of Business, İstanbul Medipol University, Turkey; c Department of Economics and Management, Khazar University, Baku, Azerbaijan; d Information Systems Department, Faculty of Computing and Information Technology, King Abdulaziz University, Jeddah, 21589, Saudi Arabia

**Keywords:** Smart investment choices, Sustainable cities, Circular economy, Neuro decision-making, Facial expression

## Abstract

Sustainable cities are crucial in establishing effective waste management systems and minimizing environmental pollution. For cities to be sustainable, different aspects need to be considered, such as technological development, clean energy usage, and energy efficiency. However, taking the most important actions is essential because of the very high cost that will arise, and this situation causes countries to have budget deficit problems. In other words, there is a significant need for a new study that makes a priority analysis with respect to the circular economy-based criteria for smart cities. Accordingly, this study aims to identify significant factors to improve sustainable cities using a novel decision-making model. First, essential determinants of the smart cities were evaluated with the decision-making trial and evaluation laboratory (DEMATEL) technique based on quantum spherical fuzzy sets (QASH) and facial expressions of the decision-makers. Second, smart investment choices for sustainable cities were ranked according to the technique for order preference by similarity to ideal solution (TOPSIS) approach. In addition, comparative ranking results were constructed together with sensitivity analysis. The ranking results of the extended VIseKriterijumska Optimizacija I Kompromisno Resenje (VIKOR) are compared with the extended TOPSIS results and their sensitivity analysis results. The main contribution of this study is that appropriate priority strategies were determined by using an original methodology to have sustainable cities. A new methodology is developed in this study by the name of neuro decision-making. According to the comparative evaluation and sensitivity analysis, the findings are found as reliable and relevant. Resource efficiency is the most critical factor in improving sustainable cities. Constructing sustainable buildings is the most appropriate strategy for increasing smart cities. Necessary actions should be taken to minimize unconscious water and energy use. New technological developments need to be quickly adapted to businesses. In this way, it would be possible to perform the same work amount using less energy and water. For this purpose, it is important both to provide the necessary training and to emphasize the importance of these issues in television advertisements.

## Highlights

• This study aims to identify significant factors to improve sustainable cities.
• Essential determinants of the smart cities are evaluated with DEMATEL.
• Smart investment choices are ranked by TOPSIS.
• Comparative ranking results are constructed by VIKOR.
• Resource efficiency is the most critical factor.
• New technological developments need to be quickly adapted.

## Teaser

In this study, appropriate priority strategies are determined to have sustainable cities so that environmental pollution can be decreased effectively.

## 1 Introduction

Sustainable cities refer to living spaces where the environment is polluted to a minimum and resources are used effectively and efficiently. In these cities, it is aimed to consume natural resources in small amounts. In addition, establishing regional waste management systems is one of the important features of these cities [1]. On the other hand, people living in these cities should be highly aware of environmental factors [2]. Due to this situation, sustainable cities refer to more livable regions [3]. Some issues need to be considered to have sustainable cities. A significant majority of these issues are closely related to circular economy goals. The circular economy is basically based on the recycling of the production, use, and disposal process of products [4]. In this context, to achieve sustainable city goals, primarily, less energy should be consumed. Thus, it is important to take some actions to ensure energy efficiency [5]. In addition to this situation, using clean energy also plays a vital role in this process. In this way, environmental pollution will be minimized, contributing to cities' sustainability [6].

Many different circular economy-based actions can be taken for cities to be sustainable. For instance, digital infrastructure plays an important role in sustainable cities. Due to the developing technology, offering innovative solutions will be much easier. It will be possible to take the specified actions thanks to the use of digital systems. Another aspect necessary for sustainable cities is the collaborative governance [7]. With the help of the integrated work of these segments, it will be possible for cities to be more sustainable. This integration enables the problems occurring in cities to be detected early and necessary measures to be taken promptly. This will help solve problems, such as unconscious natural resource consumption and environmental pollution [8]. However, these actions also create costs for countries. In this context, taking the most important actions is important. Otherwise, a very high cost will arise that causes countries to have budget deficit problems. In summary, the most important actions should be determined. However, in the literature, there are limited studies that focused on the important circular economy-based criteria for smart cities. Therefore, it is seen that there is a serious need for academic studies in which priority analysis will be made among these criteria to achieve this goal [9]. The results of this study pave the way for the investors to make strategic decisions.

Accordingly, this study aimed to identify critical issues to generate sustainable cities. Within this scope, a two-stage decision-making model was generated. First, critical indicators of the smart cities are evaluated with the decision-making trial and evaluation laboratory (DEMATEL) technique based on quantum spherical fuzzy sets (QASH) and the facial expressions of the decision-makers. Second, smart investment choices for sustainable cities were ranked with the technique for order preference by similarity to ideal solution (TOPSIS) approach. Comparative ranking results were also constructed together with sensitivity analysis. The ranking results of the extended VIseKriterijumska Optimizacija I Kompromisno Resenje (VIKOR) were compared with the extended TOPSIS results and their sensitivity analysis results. The main motivation of this study is the need for a comprehensive evaluation to define smart investment choices for sustainable cities. In the literature, different techniques were considered for this purpose, such as balanced scorecard. Decision-making techniques can also be used in this framework. However, existing techniques are also criticized because of some reasons. Hence, in the process of generating new model, these criticisms should be overcome. Uncertainty is the main problem in the decision-making evaluations. Since the problems are very complex, it is very difficult to find optimal solutions in this process. In other words, to achieve the most appropriate results, the uncertainty in the process must be managed effectively. Otherwise, the results obtained will not fully express the truth. The main superiorities of the proposed model are indicated as follows. (1) Integrating facial expression and quantum mechanisms into decision-making techniques also increases the superiority of the developed model. By examining facial expressions, the emotions of decision-makers can be taken into consideration. This situation will provide an idea about the questions that remain in doubt. Thus, it will minimize the uncertainties in the problem-solving process. The quantum mechanism will also help increase the model's success as it considers different possibilities. (2) Considering QASH, DEMATEL, and TOPSIS approaches also provide some advantages. The hesitancy situation can be considered using QASH. This will ensure that the results obtained will be more reliable. On the other hand, owing to the DEMATEL technique, the causal relationship between the indicators will also be determined. A comparative evaluation was performed using the VIKOR methodology, and a sensitivity analysis was made by considering six different cases. This situation provided an opportunity to test the validity and coherency of the proposed model.

The next section reviews the literature. The methodology is explained in the following section. The other three sections include analysis results, discussions, and conclusions.

## 2 Literature review

In this section, a literature review is conducted for circular economy criteria in sustainable smart cities. In this context, studies on each variable are summarized in separate paragraphs. For cities to be sustainable, the energy used must be clean. Liu et al. [10] stated that the preference for fossil fuels in energy production creates significant air pollution. This situation affects the country negatively in many aspects, such as the increase in chest diseases and the pollution of natural resources. In summary, using fossil fuels reduces the possibility of cities being sustainable [11]. Brown et al. [12] defined that renewable energy sources are considered to achieve sustainable city goals. Reducing air pollution is essential for cities to be sustainable. In this regard, the green energy source choice helps to achieve this goal [13]. Therefore, necessary improvements need to be made to address the weaknesses of renewable energy projects [14,15]. Kolade et al. [16] identified that a competent workforce is needed to increase these projects. It is important to provide the necessary training to the personnel. Thanks to government support, reducing the costs of green energy projects will also contribute to the increase in these projects.

Less natural resource consumption also helps to increase sustainable cities. Zeng and Zhang [17] and Figge and Thorpe [18] identified that due to unconscious natural resource consumption, problems such as global warming occur, which are very effective in the world. This situation creates differences in climatic conditions. As a result, many sectors are adversely affected [19]. On the other hand, for cities to be sustainable, less natural resource consumption should be ensured [20]. Christensen [13] and Arfaoui et al. [7] stated that particular attention should be paid to energy efficiency in this process. Machines using new technology should be preferred. In this way, it will be possible to perform the same work with less electricity consumption [21]. Berardi and de Brito [22] explained that giving energy-saving training to people living in cities is necessary. According to Schultz et al. [23], owing to this training, the awareness level of people will increase, and this will help to consume less energy.

The technological infrastructure is another important issue for cities to be sustainable. Kolade et al. (2022) emphasized that developing technology leads to a decrease in damage to environmental factors. Mangers et al. (2023) underlined that the costs of clean energy projects can be reduced thanks to developing technology. On the other hand, Kofos et al. (2022) discussed that developing technology also enables fossil fuels to be used in an environmentally friendly way. Carbon capture technology prevents the carbon gas emission formed due to fossil fuel use into the atmosphere. Kurniawan et al. (2022) stated that with smart systems, it would be easier to increase clean energy projects. According to Silvestri et al. (2021), the excess energy produced is automatically delivered to the segments needing energy due to the digital systems developed in these projects. This situation helps to eliminate imbalances in the electricity amount in clean energy projects [24]. Hosseini-Motlagh et al. [25] mentioned that with the technological infrastructure development, it would be possible to reduce energy storage costs. This issue will contribute positively to the increase in renewable energy investments.

Integration between the state, industry, and the public is also crucial for cities to be sustainable. Nian et al. [26] stated that this situation is necessary for the early detection of problems. Problems detected at an early level also allow for quick action. Bao and Lu [27] identified that this contributes to achieving the sustainable city goal. On the other hand, this integration also helps to reach effective solution suggestions for problems. Furthermore, Geraedts [28] and Xue et al. [29] concluded that this integration also makes the country resilient to social and economic risks. In an environment where the state successfully communicates with the public and companies, it will be possible to detect possible risks. Similarly, according to Christensen [13], it is only possible with this integration to reach solution-oriented ideas about the measures that can be taken against these risks. If these risks cannot be managed effectively, it will be challenging to ensure the sustainability of cities.

The main results of the literature review are demonstrated as follows. The popularity of sustainable cities is increasing. For cities to be sustainable, different aspects need to be considered, such as technological development, clean energy usage, and energy efficiency. The actions to improve these factors create some costs. Thus, the most significant indicators should be identified because of the budget constraints. However, only a limited number of studies have made a priority analysis, so there is a strong need for this type of study. While considering these issues, this study aimed to identify critical issues to generate sustainable cities by considering a novel model.

## 3 Methodology

A novel model is constructed to find significant determinants of sustainable cities that has two different parts. In the first stage, selected criteria are weighted via the extension of DEMATEL. For this purpose, facial expressions of the experts are taken into consideration while taking the evaluations from these people. Additionally, these evaluations are also converted into quantum spherical fuzzy sets to minimize uncertainties. In the final stage, smart investment choices for sustainable cities are also ranked by using TOPSIS. The details of facial expressions, quantum-based fuzzy sets, DEMATEL and TOPSIS are explained in this section.

### 3.1 Decision-making with facial expressions

One of the most important issues when creating decision-making models is correctly providing expert opinions. In this process, the experts must have knowledge of the subject. However, even if they are qualified, experts may be hesitant to answer some questions. Facial expressions consider the emotions of decision-makers in this process. In this framework, a novel methodology is developed with the name of neuro decision-making. With the help of this issue, the nonverbal emotions of the experts were analyzed, such as surprise, happiness, and intermediate emotions [30]. Similarly, Ekman and Friesen [31] generated The Facial Action Coding System (FACS) to identify nonverbal behaviors. They used 46 different action units (AUs) to evaluate these behaviors effectively.

### 3.2 Quantum-based fuzzy sets with golden ratio

Quantum theory considers different probabilities in the analysis process [32]. Because of this advantage, this model integrates fuzzy sets into this theory. In this process, the phase angle (θ), event (u), collection (ς) and amplitude-based result ($\varphi^{2}$) are considered. This theory is explained by Equations (1), (2), (3).

(1) $$Q\left(|u>\right)=\varphi e^{j\theta }$$

(2) $$|\varsigma >=\left\{|u_{1}>,|u_{2}>,\ldots ,|u_{n}>\right\}$$

(3) $$\sum_{\left|u>\subseteq \right|\varsigma >\rangle }\left|Q\left(|u>\right)\right|=1$$

Spherical fuzzy sets (A) were created to minimize uncertainty problems in this process. The biggest advantage is that these sets consider the hesitancy conditions. They are explained in Equations (4) and (5), while μ, ν, and π denote membership, non-membership, and hesitancy parameters, respectively.

(4) $${\tilde{A}}_{S}=\left\{\langle u,(\mu_{{\tilde{A}}_{S}}\left(u\right),v_{{\tilde{A}}_{S}}\left(u\right),h_{{\tilde{A}}_{S}}\left(u\right))|u\in U\right\}$$

(5) $$0{\leq \mu }_{{\tilde{A}}_{S}}^{2}\left(u\right)+v_{{\tilde{A}}_{S}}^{2}\left(u\right)+h_{{\tilde{A}}_{S}}^{2}\left(u\right)\leq 1,\forall_{u}\in U$$

Equations (6), (7), (8) include the integration of these numbers into quantum theory.

(6) $$|\varsigma_{{\tilde{A}}_{S}}>=\left\{\langle u,(\varsigma_{\mu_{{\tilde{A}}_{S}}}\left(u\right),\varsigma_{v_{{\tilde{A}}_{S}}}\left(u\right),\varsigma_{h_{{\tilde{A}}_{S}}}\left(u\right))|u\in 2^{|\varsigma_{{\tilde{A}}_{S}}>}\right\}$$

(7) $$\varsigma =\left[\varsigma_{\mu }.e^{j2\pi .\alpha },\varsigma_{v}.e^{j2\pi .\gamma },\varsigma_{h}.e^{j2\pi .\beta }\right]$$

(8) $$\varphi^{2}=\left|\varsigma_{\mu }\left(|u_{i}>\right)\right|$$

The golden ratio (G) is considered to calculate these degrees in this proposed model. Equations (9), (10), (11), (12), (13), (14), (15) give information about these details.

(9) $$G=\frac{a}{b}$$

(10) $$G=\frac{1+\sqrt{5}}{2}=1.618\ldots$$

(11) $$\varsigma_{v}=\frac{\varsigma_{\mu }}{G}$$

(12) $$\varsigma_{h}=1-\varsigma_{\mu }-\varsigma_{v}$$

(13) $$\alpha =\left|\varsigma_{\mu }\left(|u_{i}>\right)\right|$$

(14) $$\gamma =\frac{\alpha }{G}$$

(15) β=1−α−γ

Equations (16), (17), (18), (19) focus on the operations.

(16) $$\lambda *{\tilde{A}}_{\varsigma }=\left\{{\left(1-{\left(1-{\varsigma_{\mu_{\tilde{A}}}}^{2}\right)}^{\lambda }\right)}^{\frac{1}{2}}e^{j2\pi .{\left(1-{\left(1-{\left(\frac{\alpha_{\tilde{A}}}{2\pi }\right)}^{2}\right)}^{\lambda }\right)}^{\frac{1}{2}}},{\varsigma_{v_{\tilde{A}}}}^{\lambda }e^{j2\pi .{\left(\frac{\gamma_{\tilde{A}}}{2\pi }\right)}^{\lambda }},{\left({\left(1-{\varsigma_{h_{\tilde{A}}}}^{2}\right)}^{\lambda }-{\left(1-{\varsigma_{\mu_{\tilde{A}}}}^{2}-{\varsigma_{h_{\tilde{A}}}}^{2}\right)}^{\lambda }\right)}^{\frac{1}{2}}e^{j2\pi .{\left({\left(1-{\left(\frac{\beta_{\tilde{A}}}{2\pi }\right)}^{2}\right)}^{\lambda }-{\left(1-{\left(\frac{\alpha_{\tilde{A}}}{2\pi }\right)}^{2}-{\left(\frac{\beta_{\tilde{A}}}{2\pi }\right)}^{2}\right)}^{\lambda }\right)}^{\frac{1}{2}}}\right\},\lambda >0$$

(17) $${{\tilde{A}}_{\varsigma }}^{\lambda }=\left\{{\varsigma_{\mu_{\tilde{A}}}}^{\lambda }e^{j2\pi .{\left(\frac{\alpha_{\tilde{A}}}{2\pi }\right)}^{\lambda }},{\left(1-{\left(1-{\varsigma_{v_{\tilde{A}}}}^{2}\right)}^{\lambda }\right)}^{\frac{1}{2}}e^{j2\pi .{\left(1-{\left(1-{\left(\frac{\gamma_{\tilde{A}}}{2\pi }\right)}^{2}\right)}^{\lambda }\right)}^{\frac{1}{2}}},{\left({\left(1-{\varsigma_{v_{\tilde{A}}}}^{2}\right)}^{\lambda }-{\left(1-{\varsigma_{v_{\tilde{A}}}}^{2}-{\varsigma_{h_{\tilde{A}}}}^{2}\right)}^{\lambda }\right)}^{\frac{1}{2}}e^{j2\pi .{\left({\left(1-{\left(\frac{\gamma_{\tilde{A}}}{2\pi }\right)}^{2}\right)}^{\lambda }-{\left(1-{\left(\frac{\gamma_{\tilde{A}}}{2\pi }\right)}^{2}-{\left(\frac{\beta_{\tilde{A}}}{2\pi }\right)}^{2}\right)}^{\lambda }\right)}^{\frac{1}{2}}}\;\right\},\lambda >0$$

(18) $${\tilde{A}}_{\varsigma }⊕{\tilde{B}}_{\varsigma }=\left\{{\left({\varsigma_{\mu_{\tilde{A}}}}^{2}+{\varsigma_{\mu_{\tilde{B}}}}^{2}-{\varsigma_{\mu_{\tilde{A}}}}^{2}{\varsigma_{\mu_{\tilde{B}}}}^{2}\right)}^{\frac{1}{2}}e^{j2\pi .{\left({\left(\frac{\alpha_{\tilde{A}}}{2\pi }\right)}^{2}+{\left(\frac{\alpha_{\tilde{B}}}{2\pi }\right)}^{2}-{\left(\frac{\alpha_{\tilde{A}}}{2\pi }\right)}^{2}{\left(\frac{\alpha_{\tilde{B}}}{2\pi }\right)}^{2}\right)}^{\frac{1}{2}}},\varsigma_{v_{\tilde{A}}}\varsigma_{v_{\tilde{B}}}e^{j2\pi .\left(\left(\frac{\gamma_{\tilde{A}}}{2\pi }\right)\left(\frac{\gamma_{\tilde{B}}}{2\pi }\right)\right)},{\left(\left(1-{\varsigma_{\mu_{\tilde{B}}}}^{2}\right){\varsigma_{h_{\tilde{A}}}}^{2}+\left(1-{\varsigma_{\mu_{\tilde{A}}}}^{2}\right){\varsigma_{h_{\tilde{B}}}}^{2}-{\varsigma_{h_{\tilde{A}}}}^{2}{\varsigma_{h_{\tilde{B}}}}^{2}\right)}^{\frac{1}{2}}e^{j2\pi .{\left(\left(1-{\left(\frac{\alpha_{\tilde{B}}}{2\pi }\right)}^{2}\right){\left(\frac{\beta_{\tilde{A}}}{2\pi }\right)}^{2}+\left(1-{\left(\frac{\alpha_{\tilde{A}}}{2\pi }\right)}^{2}\right){\left(\frac{\beta_{\tilde{B}}}{2\pi }\right)}^{2}-{\left(\frac{\beta_{\tilde{A}}}{2\pi }\right)}^{2}{\left(\frac{\beta_{\tilde{B}}}{2\pi }\right)}^{2}\right)}^{\frac{1}{2}}}\right\}$$

(19) $${\tilde{A}}_{\varsigma }⊗{\tilde{B}}_{\varsigma }=\left\{{\varsigma_{\mu_{\tilde{A}}}\varsigma_{\mu_{\tilde{B}}}e^{j2\pi .\left(\frac{\alpha_{\tilde{A}}}{2\pi }\right)\left(\frac{\alpha_{\tilde{B}}}{2\pi }\right)},\left({\varsigma_{v_{\tilde{A}}}}^{2}+{\varsigma_{v_{\tilde{B}}}}^{2}-{\varsigma_{v_{\tilde{A}}}}^{2}{\varsigma_{v_{\tilde{B}}}}^{2}\right)}^{\frac{1}{2}}e^{j2\pi .{\left({\left(\frac{\gamma_{\tilde{A}}}{2\pi }\right)}^{2}+{\left(\frac{\gamma_{\tilde{B}}}{2\pi }\right)}^{2}-{\left(\frac{\gamma_{\tilde{A}}}{2\pi }\right)}^{2}{\left(\frac{\gamma_{\tilde{B}}}{2\pi }\right)}^{2}\right)}^{\frac{1}{2}}},{\left(\left(1-{\varsigma_{v_{\tilde{B}}}}^{2}\right){\varsigma_{h_{\tilde{A}}}}^{2}+\left(1-{\varsigma_{v_{\tilde{A}}}}^{2}\right){\varsigma_{h_{\tilde{B}}}}^{2}-{\varsigma_{h_{\tilde{A}}}}^{2}{\varsigma_{h_{\tilde{B}}}}^{2}\right)}^{\frac{1}{2}}e^{j2\pi .{\left(\left(1-{\left(\frac{\gamma_{\tilde{B}}}{2\pi }\right)}^{2}\right){\left(\frac{\beta_{\tilde{A}}}{2\pi }\right)}^{2}+\left(1-{\left(\frac{\gamma_{\tilde{A}}}{2\pi }\right)}^{2}\right){\left(\frac{\beta_{\tilde{B}}}{2\pi }\right)}^{2}-{\left(\frac{\beta_{\tilde{A}}}{2\pi }\right)}^{2}{\left(\frac{\beta_{\tilde{B}}}{2\pi }\right)}^{2}\right)}^{\frac{1}{2}}}\right\}$$

### 3.3 The extended approach to DEMATEL

DEMATEL is considered for computing the significance weights of the factors. This technique has some significant advantages, such as understanding the possible influences between criteria. With the help of this situation, an effective solution can be provided for complex relationships [33]. In this model, DEMATEL is extended by considering the following steps.

Step 1: Evaluations were taken from the expert team.
Step 2: A decision matrix is created with Equation (20).

(20) $$\varsigma_{k}=\left[\begin{array}{cccccc} 0 & \varsigma_{12} & \cdots & & \cdots & \varsigma_{1n}\\ \varsigma_{21} & 0 & \cdots & & \cdots & \varsigma_{2n}\\ \vdots & \vdots & \ddots & & \cdots & \cdots \\ \vdots & \vdots & \vdots & & \ddots & \vdots \\ \varsigma_{n1} & \varsigma_{n2} & \cdots & & \cdots & 0 \end{array}\right]$$

The aggregated values (ς) of the decision-makers are demonstrated in Equation (21).

(21) $$\varsigma =\left\{{\left[1-\prod_{i=1}^{k}{\left(1-{\varsigma_{\mu_{i}}}^{2}\right)}^{\frac{1}{k}}\right]}^{\frac{1}{2}}e^{2\pi .{\left[1-\prod_{i=1}^{k}{\left(1-{\left(\frac{\alpha_{i}}{2\pi }\right)}^{2}\right)}^{\frac{1}{k}}\right]}^{\frac{1}{2}}},\prod_{i=1}^{k}{\varsigma_{v_{i}}}^{\frac{1}{k}}e^{2\pi .\prod_{i=1}^{k}{\left(\frac{\gamma_{i}}{2\pi }\right)}^{\frac{1}{k}}},{\left[\prod_{i=1}^{k}{\left(1-{\varsigma_{\mu_{i}}}^{2}\right)}^{\frac{1}{k}}-\prod_{i=1}^{k}{\left(1-{\varsigma_{\mu_{i}}}^{2}-{\varsigma_{h_{i}}}^{2}\right)}^{\frac{1}{k}}\right]}^{\frac{1}{2}}e^{2\pi .{\left[\prod_{i=1}^{k}{\left(1-{\left(\frac{\alpha_{i}}{2\pi }\right)}^{2}\right)}^{\frac{1}{k}}-\prod_{i=1}^{k}{\left(1-{\left(\frac{\alpha_{i}}{2\pi }\right)}^{2}-{\left(\frac{\beta_{i}}{2\pi }\right)}^{2}\right)}^{\frac{1}{k}}\right]}^{\frac{1}{2}}}\right\}$$

Step 3: Defuzzified values (Defς) are computed with Equation (22).

(22) $${\mathrm{Def}\;\varsigma }_{i}=\varsigma_{\mu_{i}}+\varsigma_{h_{i}}\left(\frac{\varsigma_{\mu_{i}}}{\varsigma_{\mu_{i}}+\varsigma_{v_{i}}}\right)+\left(\frac{\alpha_{i}}{2\pi }\right)+\left(\frac{\gamma_{i}}{2\pi }\right)\left(\frac{\left(\frac{\alpha_{i}}{2\pi }\right)}{\left(\frac{\alpha_{i}}{2\pi }\right)+\left(\frac{\beta_{i}}{2\pi }\right)}\right)$$

Step 4: The normalized matrix (B) is constructed by Equations (23) and (24).

(23) $$B=\frac{\varsigma }{\max_{1\leq i\leq n}\sum_{j=1}^{n}\varsigma_{ij}}$$

where, $0\leq b_{ij}\leq 1$ (24)

Step 5: The total relationship matrix (C) is generated by Equation (25).

(25) $$C=\lim_{k\to \infty }{\left(B+B^{2}+\ldots +B^{k}\right)=B\left(I-B\right)}^{-1}$$

Step 6: Equations (26), (27) are used to identify the causal directions and weights of the items.

(26) $$D={\left[\sum_{j=1}^{n}e_{ij}\right]}_{nx1}$$

(27) $$E={\left[\sum_{i=1}^{n}e_{ij}\right]}_{1xn}$$

The threshold value (a) is computed as in Equation (28) with respect to the cause and impact relationship.

(28) $$\alpha =\frac{\sum_{i=1}^{n}\sum_{j=1}^{n}\left[e_{ij}\right]}{N}$$

### 3.4 The extended approach to TOPSIS

TOPSIS is considered to find the most critical alternatives [34]. This approach is integrated with QASH in this study as follows.

Step 1: Evaluations were collected.
Step 2: The decision matrix (X) is created by Equation (29)

(29) $$X_{k}=\left[\begin{array}{cccccc} 0 & X_{12} & \cdots & & \cdots & X_{1m}\\ X_{21} & 0 & \cdots & & \cdots & X_{2m}\\ \vdots & \vdots & \ddots & & \cdots & \cdots \\ \vdots & \vdots & \vdots & & \ddots & \vdots \\ X_{n1} & X_{n2} & \cdots & & \cdots & 0 \end{array}\right]$$

Step 3: The defuzzification process is performed.
Step 4: Equation (30) is used for normalization.

(30) $$r_{ij}=\frac{X_{ij}}{\sqrt{\sum_{i=1}^{m}X_{ij}^{2}}}\text{.}$$

Step 5: Weighted values are defined by Equation (31)

(31) $$v_{ij}=w_{ij}\times r_{ij}\text{.}$$

Step 6: Positive/negative ideal solutions ($A^{+}$ and $A^{-}$) are determined as in Equations (32), (33).

(32) $$A^{+}=\left\{v_{1j},v_{2j},\ldots ,v_{mj}\right\}=\left\{\max\;v_{1j}\;for\;\forall \;j\in n\right\}\text{,}$$

(33) $${\;A}^{-}=\left\{v_{1j},v_{2j},\ldots ,v_{mj}\right\}=\left\{\min\;v_{1j}\;for\;\forall \;j\in n\right\}\text{.}$$

Step 7: The distances to these positive and negative ideal solutions ($D_{i}^{+}$ and $D_{i}^{-}$) are calculated by Equations (34), (35).

(34) $$D_{i}^{+}=\sqrt{\sum_{j=1}^{n}{\left(v_{ij}-A_{j}^{+}\right)}^{2}}\text{,}$$

(35) $$D_{i}^{-}=\sqrt{\sum_{j=1}^{n}{\left(v_{ij}-A_{j}^{-}\right)}^{2}}\text{.}$$

Step 8: Relative closeness $\left({RC}_{i}\right)$ is created by Equation (36).

(36) $${RC}_{i}=\frac{D_{i}^{-}}{D_{i}^{+}+D_{i}^{-}}\text{.}$$

### 3.5 The extended approach to VIKOR

VIKOR is also used for an alternative ranking [35]. This method considers the closest approximation to an ideal solution. The first three steps are similar to the TOPSIS technique. Next, the best and worst values (${\tilde{f}}_{J}^{*}$ and ${\tilde{f}}_{j}^{-}$) are computed with Equation (37).

(37) $${\tilde{f}}_{J}^{*}=\max_{i}{\tilde{x}}_{ij},\text{and}\;{\tilde{f}}_{j}^{-}=\min_{i}{\tilde{x}}_{ij}\text{,}$$

In the following step, the mean group utility and maximal regret are calculated by Equations (38), (39).

(38) $${\tilde{S}}_{i}=\sum_{i=1}^{n}{\tilde{w}}_{j}\frac{\left(\left|{{\tilde{f}}_{j}}^{*}-{\tilde{x}}_{ij}\right|\right)}{\left(\left|{{\tilde{f}}_{j}}^{*}-{{\tilde{f}}_{j}}^{-}\right|\right)}$$

(39) $${\tilde{R}}_{i}=\max_{j}\left[{\tilde{w}}_{j}\frac{\left(\left|{{\tilde{f}}_{j}}^{*}-{\tilde{x}}_{ij}\right|\right)}{\left(\left|{{\tilde{f}}_{j}}^{*}-{{\tilde{f}}_{j}}^{-}\right|\right)}\right]$$

After that, Equation (40) is used for the final ranking of alternatives.

(40) $${\tilde{Q}}_{i}=v\left({\tilde{S}}_{i}-{\tilde{S}}^{*}\right)/\left({\tilde{S}}^{-}-{\tilde{S}}^{*}\right)+\left(1-v\right)\left({\tilde{R}}_{i}-{\tilde{R}}^{*}\right)/\left({\tilde{R}}^{-}-{\tilde{R}}^{*}\right)$$

## 4 An evaluation with a hybrid decision-making model

The details of the evaluation are presented in the following subtitles.

### 4.1 The importance of the problem

The sustainable city concept has become popular, especially in the last few years. For cities to be sustainable, different aspects need to be considered, such as technological development, clean energy usage, and energy efficiency. Nonetheless, the actions for improving these factors create some costs. Hence, the most significant indicators should be identified because of the budget constraints. It is seen that there is a significant need for a new study that makes a priority analysis among these criteria. By considering these issues, this study aimed to identify critical issues to generate sustainable cities by considering a novel model.

### 4.2 The details of the proposed model

**Fig. 1:**
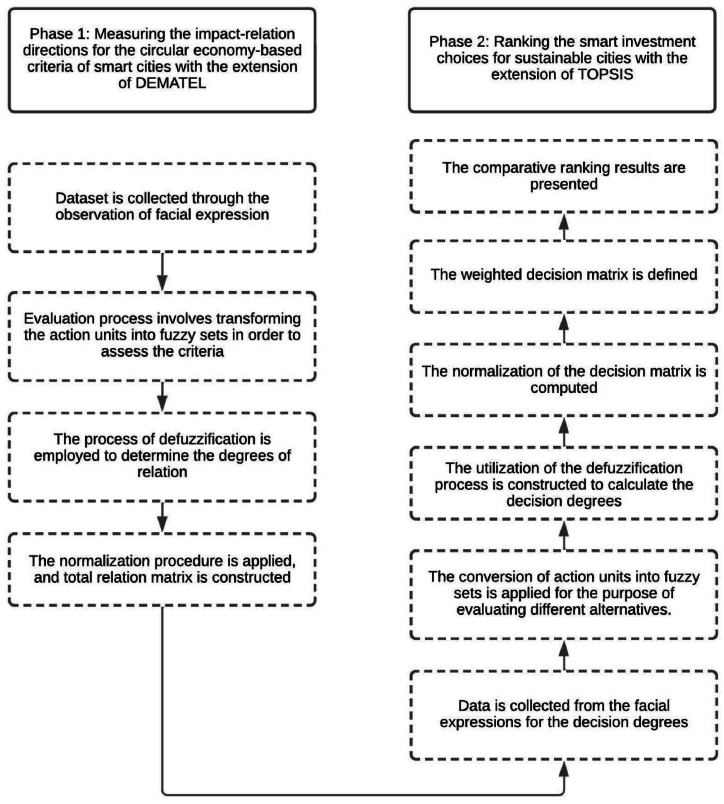
Flowchart.

A novel model was created to find significant determinants of sustainable cities. This model consisted of two different stages. In the beginning, the impact-relation directions for the circular economy-based criteria of smart cities are measured with the DEMATEL extension. There are lots of methodologies in the literature to calculate the weights of the criteria, such as AHP and ANP. However, the main superiority of DEMATEL is that causal directions between the factors can be identified [36]. The determinants of the sustainable cities can make an impact on each other. Because of this condition, using DEMATEL methodology provides an opportunity to reach more appropriate findings. Second, smart investment choices for sustainable cities are ranked with the TOPSIS extension. The main advantage of TOPSIS is that the distances to both the positive and negative solutions can be taken into consideration [37]. With the help of this superiority, TOPSIS technique helps to reach more appropriate solutions. In this process, quantum-based fuzzy sets with golden ratio and facial expressions are also taken into consideration. Moreover, quantum theory is mainly used in the physics to make more effective estimations. Owing to this benefit, this theory is integrated into the fuzzy logic to minimize uncertainty. In addition to them, considering facial expressions of the experts can have a positive contribution to the effectiveness of the proposed model. In this way, the hesitancies experienced by experts while answering questions can be taken into more consideration [38]. This situation allows more appropriate results to be achieved. The details of this model are illustrated in Fig. 1.

### 4.3 Defining selected criteria and alternatives

An expert team is created with three different people. Two of them work as a general manager in international energy investment companies. They conducted many different projects, such as smart cities, clean energy investments, energy storage process and efficiency in green energy investments. On the other side, the third expert is an academician that published many different significant papers related to the energy investments and smart cities. These people have minimum 31 years of working experience. The criteria and the alternatives for the smart investment choices of sustainable cities are listed by the supported literature in Table 1, Table 2, respectively.

**Table 1:** Circular economy-based criteria for smart cities.

Criteria
Resource efficiency (RE)
Closed-loop systems (CLS)
Collaborative governance (CG)
Digital infrastructure (DI)
Inclusivity (I)
Adaptive capacity (AC)

**Table 2:** Smart investment choices for sustainable cities.

Choices
Waste management (WST)
Green transportation (GRN)
Community engagement (COM)
Climate resilience (CLM)
Water management (WTR)
Sustainable buildings (SUS)
Renewable energy (REN)

Resource efficiency gives information about resource utilization methods, such as recycling. Closed-loop systems were designed to reduce waste by supporting reuse. Collaborative governance includes collaboration between government, businesses, and citizens to implement sustainable solutions. Digital infrastructure refers to the technological development that contributes to the circular economy. Inclusivity focuses on moving to the benefits of the circular economy. Adaptive capacity (AC) explains changing the capacity based on different circumstances.

WST refers to the effective disposal of waste generated. GRN describes how to pay attention to environmental issues in the transportation process. COM stands for agreements made by the community on environmental issues. CLM refers to projects that minimize negative developments in climatic conditions. WTR includes effective and efficient water use. SUS defines attention to energy saving in building construction. REN provides information on investments in renewable energy projects.

### 4.4 Finding the weights of the indicators

The dataset is collected through the observation of facial expressions. The dataset encompasses a range of emotions, corresponding pairs of AUs, linguistic scales, and possibility degrees, which were acquired through QASH, as depicted in Table 3.

**Table 3:** Emotional expressions and action unit combinations for linguistic and QASH.

Emotions	Selected AUs	Pair combinations of AUs	Scales for Criteria	Scales for Alternatives	Possibility Degrees	QASH
Contempt (Disdain)	7, 10, 14, 15	(7, 10), (7, 14), (7, 15), (10, 14), (10, 15), (14, 15)	No (n)	Weakest (w)	0.40	$\left[\begin{array}{c} \sqrt{0.16}e^{j2\pi .0.4}\text{,}\\ \sqrt{0.10}e^{j2\pi .0.25}\text{,}\\ \sqrt{0.74}e^{j2\pi .0.35} \end{array}\right]$
Intermediate Emotion	1 AU of Contempt +1 AU of Surprise	(7, 1), (7, 2), (7, 5), (7, 27), (10, 1), (10, 2), (10, 5), (10, 27), (14, 1), (14, 2), (14, 5), (14, 27), (15, 1), (15, 2), (15, 5), (15, 27)	some (s)	Poor (p)	0.45	$\left[\begin{array}{c} \sqrt{0.20}e^{j2\pi .0.45}\text{,}\\ \sqrt{0.13}e^{j2\pi .0.28}\text{,}\\ \sqrt{0.67}e^{j2\pi .0.27} \end{array}\right]$
Surprise	1, 2, 5, 27 1 AU of Contempt +1 AU of Happy	(1, 2), (1, 5), (1, 27), (2, 5), (2, 27), (5, 27) (7, 6), (7, 12), (7, 25), (7, 26), (10, 6), (10, 12), (10, 25), (10, 26), (14, 6), (14, 12), (14, 25), (14, 26), (15, 6), (15, 12), (15, 25), (15, 26)	medium (m)	Fair (f)	0.50	$\left[\begin{array}{c} \sqrt{0.25}e^{j2\pi .0.50}\text{,}\\ \sqrt{0.15}e^{j2\pi .0.31}\text{,}\\ \sqrt{0.60}e^{j2\pi .0.19} \end{array}\right]$
Intermediate Emotion	1 AU of Surprise +1 AU of Happy	(1, 6), (1, 12), (1, 25), (1, 26), (2, 6), (2, 12), (2, 25), (2, 26), (5, 6), (5, 12), (5, 25), (5, 26), (27, 6), (27, 12), (27, 25), (27, 26)	high (h)	Good (g)	0.55	$\left[\begin{array}{c} \sqrt{0.30}e^{j2\pi .0.55}\text{,}\\ \sqrt{0.19}e^{j2\pi .0.34}\text{,}\\ \sqrt{0.51}e^{j2\pi .0.11} \end{array}\right]$
Happiness	6, 12, 25, 26	(6, 12), (6, 25), (6, 26), (12, 25), (12, 26), (25, 26)	very high (vh)	Best (b)	0.60	$\left[\begin{array}{c} \sqrt{0.36}e^{j2\pi .0.6}\text{,}\\ \sqrt{0.22}e^{j2\pi .0.37}\text{,}\\ \sqrt{0.42}e^{j2\pi .0.03} \end{array}\right]$

The emotional expressions of the expert are analyzed with FACS. Three primary emotions (contempt, surprise, and happiness) are considered, each with distinct AUs that served as a means of quantifying the evaluations of decision-makers. The expert that observes the facial expressions of the decision-makers documents their emotional responses to criteria and alternatives using the selected AUs, which correspond to the emotions on a 5-point scale. The observer will identify the two most prominent AUs for each pairwise comparison of criteria and alternatives. If the observed AUs correspond to different emotions, the evaluation is assigned an intermediate emotion. If both contempt and happiness are observed, the evaluation is considered neutral and assigned the medium surprise scale. Observation results are given in Table 4.

**Table 4:** Observation results of facial expressions for the relation degrees.

DMKR1						
	RE	CLS	CG	DI	I	AC
RE		(6,26)	(10,26)	(5,6)	(15,12)	(12,26)
CLS	(12,26)		(15,26)	(27,25)	(15,26)	(15,26)
CG	(15,12)	(10,26)		(27,25)	(5,6)	(5,6)
DI	(5,6)	(27,25)	(7,12)		(7,12)	(2,12)
I	(15,12)	(15,26)	(27,25)	(5,27)		(2,12)
AC	(27,25)	(27,25)	(27,25)	(5,27)	(15,12)	

**DMKR2**						
	**RE**	**CLS**	**CG**	**DI**	**I**	**AC**

RE		(27,26)	(10,27)	(27,26)	(10,12)	(27,26)
CLS	(27,26)		(10,12)	(27,12)	(10,27)	(15,5)
CG	(10,27)	(15,5)		(27,12)	(10,12)	(10,12)
DI	(14,26)	(14,26)	(14,26)		(15,5)	(14,26)
I	(15,5)	(7,12)	(27,12)	(15,5)		(7,12)
AC	(27,26)	(27,12)	(27,26)	(7,12)	(10,27)	

**DMKR3**						
	**RE**	**CLS**	**CG**	**DI**	**I**	**AC**

RE		(27,12)	(10,2)	(27,25)	(27,25)	(27,12)
CLS	(27,12)		(14,26)	(5,26)	(14,27)	(10,2)
CG	(7,27)	(7,27)		(27,25)	(14,12)	(14,12)
DI	(14,27)	(14,26)	(14,26)		(7,27)	(7,27)
I	(10,2)	(5,26)	(5,26)	(10,2)		(27,12)
AC	(27,12)	(27,25)	(27,25)	(14,12)	(10,2)	

The aggregated values of the fuzzy sets were then calculated using Equation (19), as presented in Table 5.

**Table 5:** The aggregated values of QFNSs for relation degrees.

	RE	CLS	CG	DI	I	AC
RE		$\left[\begin{array}{c} \sqrt{0.32}e^{j2\pi .0.56}\text{,}\\ \sqrt{0.19}e^{j2\pi .0.34}\text{,}\\ \sqrt{0.50}e^{j2\pi .0.11} \end{array}\right]$	$\left[\begin{array}{c} \sqrt{0.22}e^{j2\pi .0.47}\text{,}\\ \sqrt{0.13}e^{j2\pi .0.29}\text{,}\\ \sqrt{0.65}e^{j2\pi .0.25} \end{array}\right]$	$\left[\begin{array}{c} \sqrt{0.30}e^{j2\pi .0.55}\text{,}\\ \sqrt{0.19}e^{j2\pi .0.34}\text{,}\\ \sqrt{0.51}e^{j2\pi .0.11} \end{array}\right]$	$\left[\begin{array}{c} \sqrt{0.27}e^{j2\pi .0.52}\text{,}\\ \sqrt{0.16}e^{j2\pi .0.32}\text{,}\\ \sqrt{0.57}e^{j2\pi .0.18} \end{array}\right]$	$\left[\begin{array}{c} \sqrt{0.32}e^{j2\pi .0.56}\text{,}\\ \sqrt{0.19}e^{j2\pi .0.34}\text{,}\\ \sqrt{0.50}e^{j2\pi .0.11} \end{array}\right]$
CLS	$\left[\begin{array}{c} \sqrt{0.32}e^{j2\pi .0.56}\text{,}\\ \sqrt{0.19}e^{j2\pi .0.34}\text{,}\\ \sqrt{0.50}e^{j2\pi .0.11} \end{array}\right]$		$\left[\begin{array}{c} \sqrt{0.25}e^{j2\pi .0.50}\text{,}\\ \sqrt{0.15}e^{j2\pi .0.31}\text{,}\\ \sqrt{0.60}e^{j2\pi .0.19} \end{array}\right]$	$\left[\begin{array}{c} \sqrt{0.30}e^{j2\pi .0.55}\text{,}\\ \sqrt{0.19}e^{j2\pi .0.34}\text{,}\\ \sqrt{0.51}e^{j2\pi .0.11} \end{array}\right]$	$\left[\begin{array}{c} \sqrt{0.22}e^{j2\pi .0.47}\text{,}\\ \sqrt{0.13}e^{j2\pi .0.29}\text{,}\\ \sqrt{0.65}e^{j2\pi .0.25} \end{array}\right]$	$\left[\begin{array}{c} \sqrt{0.22}e^{j2\pi .0.47}\text{,}\\ \sqrt{0.13}e^{j2\pi .0.29}\text{,}\\ \sqrt{0.65}e^{j2\pi .0.25} \end{array}\right]$
CG	$\left[\begin{array}{c} \sqrt{0.22}e^{j2\pi .0.47}\text{,}\\ \sqrt{0.13}e^{j2\pi .0.29}\text{,}\\ \sqrt{0.65}e^{j2\pi .0.25} \end{array}\right]$	$\left[\begin{array}{c} \sqrt{0.22}e^{j2\pi .0.47}\text{,}\\ \sqrt{0.13}e^{j2\pi .0.29}\text{,}\\ \sqrt{0.65}e^{j2\pi .0.25} \end{array}\right]$		$\left[\begin{array}{c} \sqrt{0.30}e^{j2\pi .0.55}\text{,}\\ \sqrt{0.19}e^{j2\pi .0.34}\text{,}\\ \sqrt{0.51}e^{j2\pi .0.11} \end{array}\right]$	$\left[\begin{array}{c} \sqrt{0.27}e^{j2\pi .0.52}\text{,}\\ \sqrt{0.16}e^{j2\pi .0.32}\text{,}\\ \sqrt{0.57}e^{j2\pi .0.18} \end{array}\right]$	$\left[\begin{array}{c} \sqrt{0.27}e^{j2\pi .0.52}\text{,}\\ \sqrt{0.16}e^{j2\pi .0.32}\text{,}\\ \sqrt{0.57}e^{j2\pi .0.18} \end{array}\right]$
DI	$\left[\begin{array}{c} \sqrt{0.26}e^{j2\pi .0.51}\text{,}\\ \sqrt{0.15}e^{j2\pi .0.31}\text{,}\\ \sqrt{0.61}e^{j2\pi .0.22} \end{array}\right]$	$\left[\begin{array}{c} \sqrt{0.27}e^{j2\pi .0.52}\text{,}\\ \sqrt{0.16}e^{j2\pi .0.32}\text{,}\\ \sqrt{0.57}e^{j2\pi .0.18} \end{array}\right]$	$\left[\begin{array}{c} \sqrt{0.25}e^{j2\pi .0.50}\text{,}\\ \sqrt{0.15}e^{j2\pi .0.31}\text{,}\\ \sqrt{0.60}e^{j2\pi .0.19} \end{array}\right]$		$\left[\begin{array}{c} \sqrt{0.22}e^{j2\pi .0.47}\text{,}\\ \sqrt{0.13}e^{j2\pi .0.29}\text{,}\\ \sqrt{0.65}e^{j2\pi .0.25} \end{array}\right]$	$\left[\begin{array}{c} \sqrt{0.26}e^{j2\pi .0.51}\text{,}\\ \sqrt{0.15}e^{j2\pi .0.31}\text{,}\\ \sqrt{0.61}e^{j2\pi .0.22} \end{array}\right]$
I	$\left[\begin{array}{c} \sqrt{0.22}e^{j2\pi .0.47}\text{,}\\ \sqrt{0.13}e^{j2\pi .0.29}\text{,}\\ \sqrt{0.65}e^{j2\pi .0.25} \end{array}\right]$	$\left[\begin{array}{c} \sqrt{0.27}e^{j2\pi .0.52}\text{,}\\ \sqrt{0.16}e^{j2\pi .0.32}\text{,}\\ \sqrt{0.57}e^{j2\pi .0.18} \end{array}\right]$	$\left[\begin{array}{c} \sqrt{0.30}e^{j2\pi .0.55}\text{,}\\ \sqrt{0.19}e^{j2\pi .0.34}\text{,}\\ \sqrt{0.51}e^{j2\pi .0.11} \end{array}\right]$	$\left[\begin{array}{c} \sqrt{0.22}e^{j2\pi .0.47}\text{,}\\ \sqrt{0.13}e^{j2\pi .0.29}\text{,}\\ \sqrt{0.65}e^{j2\pi .0.25} \end{array}\right]$		$\left[\begin{array}{c} \sqrt{0.29}e^{j2\pi .0.54}\text{,}\\ \sqrt{0.18}e^{j2\pi .0.33}\text{,}\\ \sqrt{0.54}e^{j2\pi .0.15} \end{array}\right]$
AC	$\left[\begin{array}{c} \sqrt{0.30}e^{j2\pi .0.55}\text{,}\\ \sqrt{0.19}e^{j2\pi .0.34}\text{,}\\ \sqrt{0.51}e^{j2\pi .0.11} \end{array}\right]$	$\left[\begin{array}{c} \sqrt{0.30}e^{j2\pi .0.55}\text{,}\\ \sqrt{0.19}e^{j2\pi .0.34}\text{,}\\ \sqrt{0.51}e^{j2\pi .0.11} \end{array}\right]$	$\left[\begin{array}{c} \sqrt{0.30}e^{j2\pi .0.55}\text{,}\\ \sqrt{0.19}e^{j2\pi .0.34}\text{,}\\ \sqrt{0.51}e^{j2\pi .0.11} \end{array}\right]$	$\left[\begin{array}{c} \sqrt{0.25}e^{j2\pi .0.50}\text{,}\\ \sqrt{0.15}e^{j2\pi .0.31}\text{,}\\ \sqrt{0.60}e^{j2\pi .0.19} \end{array}\right]$	$\left[\begin{array}{c} \sqrt{0.22}e^{j2\pi .0.47}\text{,}\\ \sqrt{0.13}e^{j2\pi .0.29}\text{,}\\ \sqrt{0.65}e^{j2\pi .0.25} \end{array}\right]$	

The defuzzification process was employed using Equation (20), as shown in Table 6.

**Table 6:** The defuzzified values.

	RE	CLS	CG	DI	I	AC
RE	.000	1.245	1.243	1.236	1.243	1.245
CLS	1.245	.000	1.236	1.236	1.243	1.243
CG	1.243	1.243	.000	1.236	1.243	1.243
DI	1.256	1.243	1.236	.000	1.243	1.256
I	1.243	1.243	1.236	1.243	.000	1.243
AC	1.236	1.236	1.236	1.236	1.243	.000

**Fig. 2:**
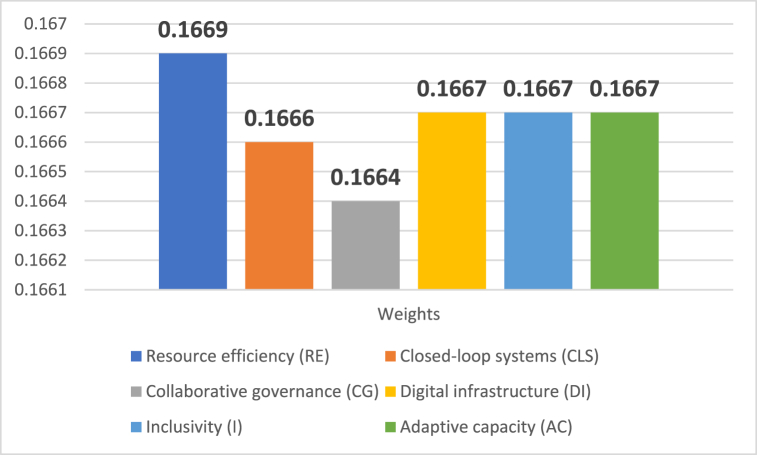
Weights of the criteria.

The normalization procedure was applied using Equations (21), (22), (23), (25), (26). After that, a total relation matrix was created. With the help of the values of these items, the weights and impact directions of the items can be identified. The weights of the criteria are demonstrated in Fig. 2.

**Fig. 3:**
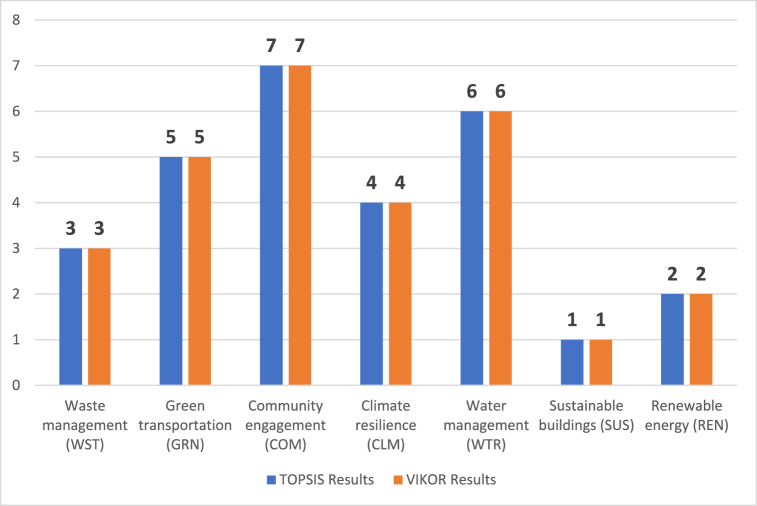
Comparative ranking results.

Digital infrastructure (DI) impacted all other criteria, while AC did not influence other circular economy-based criteria of smart cities (see Fig. 3). However, resource efficiency (RE) had the greatest weight (.1669), and collaborative governance (CG) had the weakest importance (.1664) in the criteria set.

### 4.5 Ranking the smart investment choices for sustainable cities with the extension of TOPSIS

After collecting the data regarding the alternatives, observation results were computed based on facial expressions, as presented in Table 7.

**Table 7:** Observation results of facial expressions for the decision degrees.

DMKR1						
	RE	CLS	CG	DI	I	AC
WST	(25,26)	(10,6)	(12,26)	(27,26)	(10,6)	(27,26)
GRN	(12,26)	(10,6)	(25,26)	(10,6)	(25,26)	(10,6)
COM	(12,26)	(27,26)	(15,25)	(5,25)	(10,6)	(27,26)
CLM	(25,26)	(15,25)	(5,25)	(14,25)	(2,12)	(15,25)
WTR	(25,26)	(14,25)	(27,26)	(14,25)	(27,26)	(15,25)
SUS	(27,26)	(14,25)	(5,25)	(10,6)	(2,12)	(14,25)
REN	(14,25)	(14,25)	(6,12)	(10,6)	(25,26)	(14,25)

**DMKR2**						
	**RE**	**CLS**	**CG**	**DI**	**I**	**AC**

WST	(27,12)	(14,25)	(6,25)	(2,25)	(10,6)	(27,12)
GRN	(27,26)	(15,27)	(6,25)	(10,6)	(6,25)	(14,25)
COM	(6,25)	(2,25)	(10,6)	(27,12)	(2,27)	(27,12)
CLM	(27,26)	(10,6)	(27,26)	(15,27)	(2,25)	(7,2)
WTR	(25,26)	(15,2)	(2,25)	(2,27)	(27,12)	(10,6)
SUS	(2,25)	(14,25)	(27,12)	(14,27)	(27,26)	(15,2)
REN	(7,2)	(14,27)	(2,25)	(14,25)	(2,25)	(15,27)

**DMKR3**						
	**RE**	**CLS**	**CG**	**DI**	**I**	**AC**

WST	(25,26)	(14,25)	(6,25)	(27,26)	(7,2)	(27,26)
GRN	(27,12)	(14,27)	(6,25)	(2,27)	(6,25)	(15,2)
COM	(6,25)	(27,26)	(2,27)	(27,26)	(10,6)	(27,12)
CLM	(27,12)	(14,25)	(27,12)	(7,2)	(27,12)	(14,27)
WTR	(25,26)	(7,2)	(2,25)	(15,2)	(27,12)	(14,25)
SUS	(25,26)	(15,27)	(14,27)	(7,2)	(15,27)	(7,2)
REN	(7,2)	(14,27)	(6,25)	(14,25)	(25,26)	(15,27)

Aggregated values are shown in Table 8.

**Table 8:** The aggregated values of QFNSs for decision degrees.

	RE	CLS	CG	DI	I	AC
WST	$\left[\begin{array}{c} \sqrt{0.34}e^{j2\pi .0.58}\text{,}\\ \sqrt{0.20}e^{j2\pi .0.35}\text{,}\\ \sqrt{0.47}e^{j2\pi .0.11} \end{array}\right]$	$\left[\begin{array}{c} \sqrt{0.25}e^{j2\pi .0.50}\text{,}\\ \sqrt{0.15}e^{j2\pi .0.31}\text{,}\\ \sqrt{0.60}e^{j2\pi .0.19} \end{array}\right]$	$\left[\begin{array}{c} \sqrt{0.36}e^{j2\pi .0.60}\text{,}\\ \sqrt{0.22}e^{j2\pi .0.37}\text{,}\\ \sqrt{0.42}e^{j2\pi .0.03} \end{array}\right]$	$\left[\begin{array}{c} \sqrt{0.30}e^{j2\pi .0.55}\text{,}\\ \sqrt{0.19}e^{j2\pi .0.34}\text{,}\\ \sqrt{0.51}e^{j2\pi .0.11} \end{array}\right]$	$\left[\begin{array}{c} \sqrt{0.24}e^{j2\pi .0.48}\text{,}\\ \sqrt{0.14}e^{j2\pi .0.30}\text{,}\\ \sqrt{0.62}e^{j2\pi .0.22} \end{array}\right]$	$\left[\begin{array}{c} \sqrt{0.30}e^{j2\pi .0.55}\text{,}\\ \sqrt{0.19}e^{j2\pi .0.34}\text{,}\\ \sqrt{0.51}e^{j2\pi .0.11} \end{array}\right]$
GRN	$\left[\begin{array}{c} \sqrt{0.32}e^{j2\pi .0.56}\text{,}\\ \sqrt{0.19}e^{j2\pi .0.34}\text{,}\\ \sqrt{0.50}e^{j2\pi .0.11} \end{array}\right]$	$\left[\begin{array}{c} \sqrt{0.22}e^{j2\pi .0.47}\text{,}\\ \sqrt{0.13}e^{j2\pi .0.29}\text{,}\\ \sqrt{0.65}e^{j2\pi .0.25} \end{array}\right]$	$\left[\begin{array}{c} \sqrt{0.36}e^{j2\pi .0.60}\text{,}\\ \sqrt{0.22}e^{j2\pi .0.37}\text{,}\\ \sqrt{0.42}e^{j2\pi .0.03} \end{array}\right]$	$\left[\begin{array}{c} \sqrt{0.25}e^{j2\pi .0.50}\text{,}\\ \sqrt{0.15}e^{j2\pi .0.31}\text{,}\\ \sqrt{0.60}e^{j2\pi .0.19} \end{array}\right]$	$\left[\begin{array}{c} \sqrt{0.36}e^{j2\pi .0.60}\text{,}\\ \sqrt{0.22}e^{j2\pi .0.37}\text{,}\\ \sqrt{0.42}e^{j2\pi .0.03} \end{array}\right]$	$\left[\begin{array}{c} \sqrt{0.24}e^{j2\pi .0.48}\text{,}\\ \sqrt{0.14}e^{j2\pi .0.30}\text{,}\\ \sqrt{0.62}e^{j2\pi .0.22} \end{array}\right]$
COM	$\left[\begin{array}{c} \sqrt{0.36}e^{j2\pi .0.60}\text{,}\\ \sqrt{0.22}e^{j2\pi .0.37}\text{,}\\ \sqrt{0.42}e^{j2\pi .0.03} \end{array}\right]$	$\left[\begin{array}{c} \sqrt{0.30}e^{j2\pi .0.55}\text{,}\\ \sqrt{0.19}e^{j2\pi .0.34}\text{,}\\ \sqrt{0.51}e^{j2\pi .0.11} \end{array}\right]$	$\left[\begin{array}{c} \sqrt{0.25}e^{j2\pi .0.50}\text{,}\\ \sqrt{0.15}e^{j2\pi .0.31}\text{,}\\ \sqrt{0.60}e^{j2\pi .0.19} \end{array}\right]$	$\left[\begin{array}{c} \sqrt{0.30}e^{j2\pi .0.55}\text{,}\\ \sqrt{0.19}e^{j2\pi .0.34}\text{,}\\ \sqrt{0.51}e^{j2\pi .0.11} \end{array}\right]$	$\left[\begin{array}{c} \sqrt{0.25}e^{j2\pi .0.50}\text{,}\\ \sqrt{0.15}e^{j2\pi .0.31}\text{,}\\ \sqrt{0.60}e^{j2\pi .0.19} \end{array}\right]$	$\left[\begin{array}{c} \sqrt{0.30}e^{j2\pi .0.55}\text{,}\\ \sqrt{0.19}e^{j2\pi .0.34}\text{,}\\ \sqrt{0.51}e^{j2\pi .0.11} \end{array}\right]$
CLM	$\left[\begin{array}{c} \sqrt{0.32}e^{j2\pi .0.56}\text{,}\\ \sqrt{0.19}e^{j2\pi .0.34}\text{,}\\ \sqrt{0.50}e^{j2\pi .0.11} \end{array}\right]$	$\left[\begin{array}{c} \sqrt{0.25}e^{j2\pi .0.50}\text{,}\\ \sqrt{0.15}e^{j2\pi .0.31}\text{,}\\ \sqrt{0.60}e^{j2\pi .0.19} \end{array}\right]$	$\left[\begin{array}{c} \sqrt{0.30}e^{j2\pi .0.55}\text{,}\\ \sqrt{0.19}e^{j2\pi .0.34}\text{,}\\ \sqrt{0.51}e^{j2\pi .0.11} \end{array}\right]$	$\left[\begin{array}{c} \sqrt{0.22}e^{j2\pi .0.47}\text{,}\\ \sqrt{0.13}e^{j2\pi .0.29}\text{,}\\ \sqrt{0.65}e^{j2\pi .0.25} \end{array}\right]$	$\left[\begin{array}{c} \sqrt{0.30}e^{j2\pi .0.55}\text{,}\\ \sqrt{0.19}e^{j2\pi .0.34}\text{,}\\ \sqrt{0.51}e^{j2\pi .0.11} \end{array}\right]$	$\left[\begin{array}{c} \sqrt{0.22}e^{j2\pi .0.47}\text{,}\\ \sqrt{0.13}e^{j2\pi .0.29}\text{,}\\ \sqrt{0.65}e^{j2\pi .0.25} \end{array}\right]$
WTR	$\left[\begin{array}{c} \sqrt{0.36}e^{j2\pi .0.60}\text{,}\\ \sqrt{0.22}e^{j2\pi .0.37}\text{,}\\ \sqrt{0.42}e^{j2\pi .0.03} \end{array}\right]$	$\left[\begin{array}{c} \sqrt{0.22}e^{j2\pi .0.47}\text{,}\\ \sqrt{0.13}e^{j2\pi .0.29}\text{,}\\ \sqrt{0.65}e^{j2\pi .0.25} \end{array}\right]$	$\left[\begin{array}{c} \sqrt{0.30}e^{j2\pi .0.55}\text{,}\\ \sqrt{0.19}e^{j2\pi .0.34}\text{,}\\ \sqrt{0.51}e^{j2\pi .0.11} \end{array}\right]$	$\left[\begin{array}{c} \sqrt{0.24}e^{j2\pi .0.48}\text{,}\\ \sqrt{0.14}e^{j2\pi .0.30}\text{,}\\ \sqrt{0.62}e^{j2\pi .0.22} \end{array}\right]$	$\left[\begin{array}{c} \sqrt{0.30}e^{j2\pi .0.55}\text{,}\\ \sqrt{0.19}e^{j2\pi .0.34}\text{,}\\ \sqrt{0.51}e^{j2\pi .0.11} \end{array}\right]$	$\left[\begin{array}{c} \sqrt{0.25}e^{j2\pi .0.50}\text{,}\\ \sqrt{0.15}e^{j2\pi .0.31}\text{,}\\ \sqrt{0.60}e^{j2\pi .0.19} \end{array}\right]$
SUS	$\left[\begin{array}{c} \sqrt{0.32}e^{j2\pi .0.56}\text{,}\\ \sqrt{0.19}e^{j2\pi .0.34}\text{,}\\ \sqrt{0.50}e^{j2\pi .0.11} \end{array}\right]$	$\left[\begin{array}{c} \sqrt{0.24}e^{j2\pi .0.48}\text{,}\\ \sqrt{0.14}e^{j2\pi .0.30}\text{,}\\ \sqrt{0.62}e^{j2\pi .0.22} \end{array}\right]$	$\left[\begin{array}{c} \sqrt{0.27}e^{j2\pi .0.51}\text{,}\\ \sqrt{0.16}e^{j2\pi .0.31}\text{,}\\ \sqrt{0.59}e^{j2\pi .0.20} \end{array}\right]$	$\left[\begin{array}{c} \sqrt{0.22}e^{j2\pi .0.47}\text{,}\\ \sqrt{0.13}e^{j2\pi .0.29}\text{,}\\ \sqrt{0.65}e^{j2\pi .0.25} \end{array}\right]$	$\left[\begin{array}{c} \sqrt{0.27}e^{j2\pi .0.51}\text{,}\\ \sqrt{0.16}e^{j2\pi .0.31}\text{,}\\ \sqrt{0.59}e^{j2\pi .0.20} \end{array}\right]$	$\left[\begin{array}{c} \sqrt{0.22}e^{j2\pi .0.47}\text{,}\\ \sqrt{0.13}e^{j2\pi .0.29}\text{,}\\ \sqrt{0.65}e^{j2\pi .0.25} \end{array}\right]$
REN	$\left[\begin{array}{c} \sqrt{0.22}e^{j2\pi .0.47}\text{,}\\ \sqrt{0.13}e^{j2\pi .0.29}\text{,}\\ \sqrt{0.65}e^{j2\pi .0.25} \end{array}\right]$	$\left[\begin{array}{c} \sqrt{0.22}e^{j2\pi .0.47}\text{,}\\ \sqrt{0.13}e^{j2\pi .0.29}\text{,}\\ \sqrt{0.65}e^{j2\pi .0.25} \end{array}\right]$	$\left[\begin{array}{c} \sqrt{0.34}e^{j2\pi .0.58}\text{,}\\ \sqrt{0.20}e^{j2\pi .0.35}\text{,}\\ \sqrt{0.47}e^{j2\pi .0.11} \end{array}\right]$	$\left[\begin{array}{c} \sqrt{0.25}e^{j2\pi .0.50}\text{,}\\ \sqrt{0.15}e^{j2\pi .0.31}\text{,}\\ \sqrt{0.60}e^{j2\pi .0.19} \end{array}\right]$	$\left[\begin{array}{c} \sqrt{0.34}e^{j2\pi .0.58}\text{,}\\ \sqrt{0.20}e^{j2\pi .0.35}\text{,}\\ \sqrt{0.47}e^{j2\pi .0.11} \end{array}\right]$	$\left[\begin{array}{c} \sqrt{0.22}e^{j2\pi .0.47}\text{,}\\ \sqrt{0.13}e^{j2\pi .0.29}\text{,}\\ \sqrt{0.65}e^{j2\pi .0.25} \end{array}\right]$

Defuzzified values were computed and are shown in Table 9.

**Table 9:** The defuzzified values of QFNSs for decision degrees.

	RE	CLS	CG	DI	I	AC
WST	1.247	1.236	1.236	1.236	1.243	1.236
GRN	1.245	1.243	1.236	1.236	1.236	1.243
COM	1.236	1.236	1.236	1.236	1.236	1.236
CLM	1.245	1.236	1.236	1.243	1.236	1.243
WTR	1.236	1.243	1.236	1.243	1.236	1.236
SUS	1.245	1.243	1.263	1.243	1.263	1.243
REN	1.243	1.243	1.247	1.236	1.247	1.243

Normalized values were computed by Equation (28) and are given in Table 10.

**Table 10:** The normalized decision matrix.

	RE	CLS	CG	DI	I	AC
WST	.379	.377	.376	.377	.378	.377
GRN	.379	.379	.376	.377	.376	.379
COM	.376	.377	.376	.377	.376	.377
CLM	.379	.377	.376	.379	.376	.379
WTR	.376	.379	.376	.379	.376	.377
SUS	.379	.379	.384	.379	.384	.379
REN	.378	.379	.380	.377	.379	.379

The weighted matrix was constructed by Equation (29) and is demonstrated in Table 11.

**Table 11:** The weighted decision matrix.

	RE	CLS	CG	DI	I	AC
WST	.063	.063	.063	.063	.063	.063
GRN	.063	.063	.063	.063	.062	.063
COM	.063	.063	.063	.063	.062	.063
CLM	.063	.063	.063	.063	.062	.063
WTR	.063	.063	.063	.063	.062	.063
SUS	.063	.063	.064	.063	.064	.063
REN	.063	.063	.063	.063	.063	.063

Smart investment choices were ranked using Equations (30), (31), (32), (33), (34) and are presented in Table 12.

**Table 12:** Ranking of smart investment choices.

Smart Investment Choices	D+	D-	RCi	Ranking
Waste management	.002	.001	.265	3
Green transportation	.002	.001	.256	5
Community engagement	.002	.000	.000	7
Climate resilience	.002	.001	.257	4
Water management	.002	.000	.190	6
Sustainable buildings	.000	.002	.959	1
Renewable energy	.001	.001	.450	2

Sustainable buildings (SUS) were the most appropriate strategy to increase smart cities. Renewable energy usage (REN) and effective waste management systems (WST) were the other critical alternatives. Water management (WTR) and community engagement (COM) were in the last ranks.

### 4.6 Sensitivity analysis with the extension of VIKOR

Comparative ranking results are constructed together with sensitivity analysis. The ranking results of the extended VIKOR were compared with the extended TOPSIS results and their sensitivity analysis results. There are as many different weights as there are criteria. We include their weight sets in the decision-making analysis. Therefore, 6 different ranking results are obtained. We compare them with each other. The results are presented in Table 13.

**Table 13:** Comparative ranking results of smart investment choices with sensitivity analysis.

Extended TOPSIS						
Smart Investment Choices	Case 1	Case 2	Case 3	Case 4	Case 5	Case 6
WST	3	3	3	3	3	3
GRN	5	5	4	4	5	5
COM	7	7	7	7	7	7
CLM	4	4	5	5	4	4
WTR	6	6	6	6	6	6
SUS	1	1	1	1	1	1
REN	2	2	2	2	2	2

**Extended VIKOR**						
**Smart Investment Choices**	**Case 1**	**Case 2**	**Case 3**	**Case 4**	**Case 5**	**Case 6**

WST	3	3	3	3	3	3
GRN	5	5	4	4	4	5
COM	7	7	7	7	7	7
CLM	4	4	5	5	4	4
WTR	6	6	6	6	6	6
SUS	1	1	1	1	1	1
REN	2	2	2	2	2	2

**Fig. 4:**
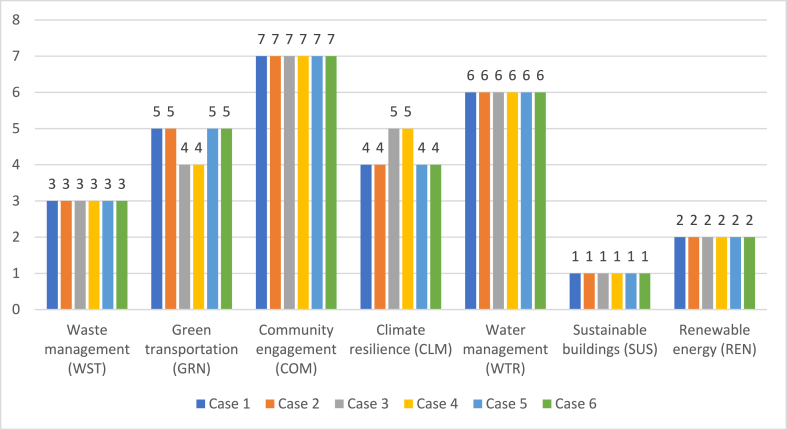
Sensitivity analysis results.

The comparative evaluation has been performed by using VIKOR methodology. Fig. 3 gives information about the comparative ranking results (see Fig. 4).

Fig. 3 gives information about the comparative evaluations. It is seen that the results of both TOPSIS and VIKOR are similar. This situation demonstrates that the proposed model provides coherent and reliable results. On the other side, a sensitivity analysis has also been conducted using six cases to validate the consistency of the criteria weights in the alternative ranking process. The criteria weights are altered consecutively, and the ranking results were repeatedly evaluated based on the changed weighting results. In addition, a comparative analysis was performed to ensure robustness. For this purpose, the QASH VIKOR method is utilized to rank the alternatives with a hybrid decision-making approach. Fig. 4 gives information about the results of the sensitivity analysis by considering 6 different cases.

Fig. 4 indicates that the results are the same for different cases. It is concluded that the ranking results obtained from both methods were coherent and applicable for further extensions.

## 5 Discussions

The sustainability of cities is vital for the social and economic development of countries. In this context, countries need to take necessary actions for sustainable urbanization. The analysis of this study showed that the resource efficiency issue is the most important. In other words, for cities to be sustainable, natural resources must be used efficiently. In this process, taking the necessary actions and bringing the consumption of resources to a reasonable level is necessary. This should not mean that other criteria are not crucial for sustainable urbanization. Instead of this situation, it is stated that resource efficiency improvements should be prioritized compared to other indicators. The water efficiency issue is important in this process. Unconscious water use causes a waste of resources. This situation leads to many social and economic problems. This condition is also underlined by many different scholars in the literature. Wei et al. [39], Li et al. [40], and Cao et al. [41] focused on this issue and claimed that water efficiency should be provided primarily for the agricultural industry. Drought problems occur because of insufficient water resources. This situation increases environmental problems. Businesses and individuals should pay attention to some issues to ensure water efficiency. Especially in agricultural areas, a lot of water is used. Shahangian et al. [42] and Freire-González [43] also recommended taking measures for individuals to save water. In this context, carrying out the necessary inspections and using only the required water amount with digital systems is necessary. In addition, it is essential to increase the awareness of individuals about water saving by providing necessary training. However, some other scholars underlined the significance of other variables. Energy efficiency is another issue to be considered in this context. There is also a natural resource depletion risk because of excessive energy consumption. Current technology must be followed seriously to ensure energy efficiency. Moreover, new technological developments need to be quickly adapted to businesses [44]. This will enable performing the same work amount using less energy. In addition, it is necessary to increase the sensitivity of individuals to the energy-saving issue [45]. For this, it is important both to provide the required training and to emphasize the importance of these issues in television advertisements. Liu et al. [46] underlined the significance of energy efficiency in using resources effectively. They recommended improving microgeneration energy technology investments to increase energy efficiency. Moreover, Temiz and Dinçer [47] developed a hybridized small modular reactor and solar-based energy system.

Sustainable buildings and renewable energy use have also been determined as the most correct strategies for sustainable urbanization. In this context, the buildings to be built should be designed to minimize the negative effects on human health and the natural environment. To achieve this goal, energy, water, and other resources should be used effectively in green buildings. In addition, renewable energy systems need to be developed in buildings. In this regard, it is essential to install solar panels in buildings. Therefore, some of the required energy will be provided by this system, and it will be possible to produce clean energy. Chatti and Khan [48] and Vainio [49] concluded that technological improvements should be prioritized to improve renewable energy systems. With the help of this situation, it can be much easier to have smart cities. Technological improvements mainly help to reduce the operation costs of the projects. This condition has a positive influence on the effectiveness of the operational procedures. Owing to this situation, financial profitability of the projects can be increased. This issue has a positive contribution to the long-run sustainability of these projects. On the other side, the importance of some other indicators is also underlined in some other studies. For instance, Bushehri et al. [50] highlighted that effective energy storage systems should be provided to increase the performance of the renewable energy investments. Renewable energy projects are negatively affected from the climate change. Gao et al. [51] determined that especially in the nights, there can be decrease in the amount of the electricity from the solar panels. Wu et al. [52] stated that to overcome this problem, energy storage projects should be improved. Smart grid technologies monitor energy flows, enabling storage systems to operate more efficiently. Similarly, Jafarizadeh et al. [53] concluded that financial incentives for energy storage projects can increase investment and increase efficiency. In this context, tax breaks can provide significant cost advantages for these projects. Analysis of energy demand and production data helps optimize storage systems.

## 6 Conclusion

This study aims to define significant factors to improve sustainable cities. A two-stage decision-making model was constructed. First, important determinants of the smart cities were evaluated using the DEMATEL technique based on QASH and the facial expressions of the decision-makers. Secondly, smart investment choices for sustainable cities are ranked by the TOPSIS approach. Additionally, comparative ranking results are constructed together with sensitivity analysis. The ranking results of the extended VIKOR are compared with the extended TOPSIS results and their sensitivity analysis results. The digital infrastructure impacted all other criteria, while AC does not influence other circular economy-based criteria of smart cities. However, resource efficiency is the most critical item, whereas collaborative governance had the weakest importance in the criteria set. Constructing sustainable buildings is the most appropriate strategy for increasing smart cities. Renewable energy usage and effective waste management systems are the other critical alternatives. The comparative evaluation and sensitivity analysis showed that the findings were reliable and relevant.

Increasing resource efficiency to achieve smart cities is an important part of creating sustainable and livable cities. To achieve this goal, it is appropriate to implement some policies and strategies. Tax reductions provided by states are an issue that should be taken into consideration in this process [54]. This allows the costs of the projects to be significantly reduced [55]. On the other hand, integrating smart meters into these projects helps increase the optimization of the projects. In addition to them, the effective waste management of these projects also enables resource efficiency to be achieved. In this context, effective waste monitoring systems should be established. Thanks to these systems, it is possible for projects to be more efficient [56,57]. Moreover, smart transportation systems should be implemented in cities. In this way, it becomes easier to optimize the traffic flow in the city. Ensuring building efficiency is another important issue in this process. In this context, new buildings need to be designed to provide energy efficiency.

The main contribution of this study is that appropriate priority strategies were determined using an original methodology to have sustainable cities. A new methodology was developed in this study by the name of neuro decision-making. However, it is also possible to mention some limitations of this study. This study presents important factors and priority strategies for increasing sustainable cities. However, no case study has been conducted regarding the accuracy of these criteria. This issue can be comprehensively addressed in future studies. On the other hand, some improvements can be made regarding the developed decision-making method. For example, it is thought that creating a more complex model by considering different fuzzy numbers will provide some benefits. In addition to this condition, in this proposed model, the evaluations of all experts are assumed with the same coefficient. However, these people can have different demographical issues. Due to this situation, in the following studies, the weights of these experts should be computed by considering their educational levels and working experiences. This issue has a positive contribution to the effectiveness of the analysis results.

## CRediT authorship contribution statement

**Gang Kou:** Writing – review & editing, Writing – original draft, Resources, Project administration, Methodology, Investigation, Funding acquisition, Formal analysis, Data curation, Conceptualization. **Hasan Dinçer:** Writing – review & editing, Writing – original draft, Resources, Methodology, Investigation, Funding acquisition, Formal analysis, Data curation, Conceptualization. **Serhat Yüksel:** Writing – review & editing, Writing – original draft, Methodology, Investigation, Funding acquisition, Formal analysis, Data curation, Conceptualization. **Fahd S. Alotaibi:** Writing – review & editing, Writing – original draft, Project administration, Methodology, Funding acquisition, Formal analysis, Data curation, Conceptualization.

## Declaration of competing interest

The authors declare that they have no known competing financial interests or personal relationships that could have appeared to influence the work reported in this paper.
